# The Associations Between Dialysis Treatment Time, Mortality, and Hospitalizations in a Large Hemodialysis Cohort

**DOI:** 10.1016/j.ekir.2026.106483

**Published:** 2026-03-14

**Authors:** Rachel Lasky, Linda H. Ficociello, Jennifer E. Flythe, Benjamin E. Hippen

**Affiliations:** 1 Renal Research Institute, Waltham, Massachusetts, USA; 2 Division of Nephrology and Hypertension, Department of Medicine, University of North Carolina School of Medicine, Chapel Hill, North Carolina, USA; 3 Global Medical Office, Fresenius Medical Care, Waltham, Massachusetts, USA

**Keywords:** hemodialysis, patient survival, treatment time

## Abstract

**Introduction:**

The relationship between hemodialysis treatment time, hospitalization rates, and mortality remains an area of controversy because of difficulties in separating the clinical effects of treatment time from urea clearance and ultrafiltration (UF) volume.

**Methods:**

Data were obtained from a retrospective cohort of 146,127 maintenance in-center hemodialysis patients, aged 18 to 89 years, who dialyzed at Fresenius Kidney Care (FKC) clinics between January 1, 2022 and July 1, 2023 with 1-year follow-up after a 30-day run-in period. The patients were stratified into 6 treatment-time groups based on their mean delivered treatment time during the exposure period (180–194, 195–209, 210–224, 225–239, 240–254, and 255–269 minutes). The primary outcome was all-cause mortality; secondary outcomes included all-cause hospitalization rates and hospital length of stay.

**Results:**

Mean dialysis vintage was > 4 years, and few patients likely had residual kidney function. Compared with individuals in the 180–194 minutes group, patients in the 240–254 minutes group had a 27% lower mortality (hazard ratio: 0.73 [0.69–0.76]), whereas patients in the 210–224 minutes and 225–239 minutes groups both had a 19% lower mortality (hazard ratio: 0.81 [0.77–0.85]) and 195–209 minutes group had 15%. These benefits were observed in patient subgroups across a wide range of mean UF volumes as well as with a spKt/V > 1.4, but not for patients with spKt/V < 1.4. In secondary analyses, similar associations were observed between longer treatment times (up to 240–254 minutes) and reduced hospitalization rates and shorter hospital stays.

**Conclusion:**

Longer dialysis treatment times are associated with better survival, fewer hospitalizations, and shorter hospital stays. Although the potential for selection bias cannot be excluded, these survival benefits were realized even when accounting for UF volume and spKt/V > 1.4.

## Graphical abstract

**Figure:**
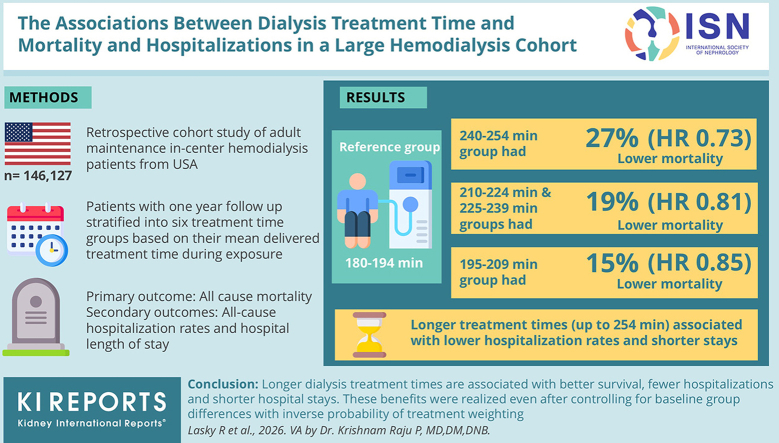

The relationship between hemodialysis treatment time and outcomes remains an area of controversy. One salient challenge is that Kt/V and UF rate, both established risk factors for adverse outcomes, are interconnected with treatment time, making it difficult to attribute potentially related outcomes to treatment time alone. The Time to Reduce Mortality in End Stage Kidney Disease trial^1^ attempted to answer the question of whether longer treatment time (≥ 255 minutes) reduces mortality and hospitalizations compared with “usual care” treatment time (typically ∼ 210 minutes). However, the trial was terminated early because of insufficient separation between treatment groups, leaving the answer an ongoing subject of debate. The inadequate uptake of the intervention in the Time to Reduce Mortality in End Stage Kidney Disease trial was attributed, in part, to patient unwillingness to undergo treatments longer than those of other patients at their clinics, underscoring the need for rigorous evidence to justify a recommendation for longer than standard treatments. In the absence of randomized controlled trials, well-designed, large observational studies play important roles in understanding the associations of treatment time and clinical outcomes.

In the current era of hemodialysis therapy, strategies to limit UF rates to < 13 ml/kg/h and reach an spKt/V > 1.2 have been implemented broadly as part of quality improvement programs aimed at meeting the thresholds of national quality performance measures. Generally, these efforts have been successful, and in 2023, <9% of individuals receiving maintenance hemodialysis in the US had UF rates > 13 ml/kg/h and >96% of individuals achieved an spKt/V > 1.2.^2^^,^^3^ This period of constrained UF rates and optimized small molecule clearance provides a unique opportunity to revisit the relationship between treatment time and mortality. The question takes on particular importance because prescribed treatments times in the US are shortening, with the proportion of prevalent hemodialysis patients with treatment times ≥ 240 minutes falling from 34.3% in 2019 to 27.9% in 2023.^4^

With this landscape in mind, we undertook this study to reexamine the associations of prescribed hemodialysis treatment time and all-cause mortality, hospitalization, and hospital days in a contemporary “post-COVID” cohort of prevalent patients receiving care at FKC clinics, a population broadly representative of the US hemodialysis population.

## Methods

**Figure 1:**
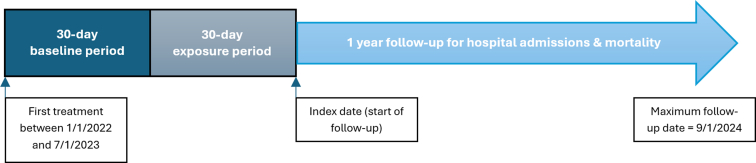
Study design.

Data were obtained from 171,863 maintenance in-center hemodialysis patients, aged 18 to 89 years, who were dialyzed at FKC clinics between January 1, 2022, and July 1, 2023. In Figure 1, we display the study design, a model like one employed by Assimon *et al.*^5^ Baseline characteristics were assessed during a 30-day baseline period, beginning on the date of a patient’s first dialysis treatment during the study period. These patient-level characteristics included age (at start of baseline), sex, race, length of time receiving hemodialysis (at start of baseline), body mass index, pre-and posthemodialysis systolic and diastolic blood pressure, estimated dry weight, primary insurance type, location of treatment facility, laboratory values, and comorbid conditions. Patients were excluded if they were discharged from FKC or died before the end of the 30-day baseline period.

The exposure, mean hemodialysis treatment time (prescribed and delivered), was assessed in a 30-day exposure period following the baseline period. Other hemodialysis treatment-related data assessed included pre- and postdialysis weight, and UF volume. Patients were excluded if they received < 7 in-center hemodialysis treatments during the exposure period or if their mean delivered treatment time was < 180 minutes or ≥ 270 minutes (Supplementary Figure S1). After the exposure period, patients were followed-up forward in historical time until death, transplantation, change in dialysis modality, recovery of kidney function, end of care at FKC, or end of 1-year follow-up.

**Figure 2:**
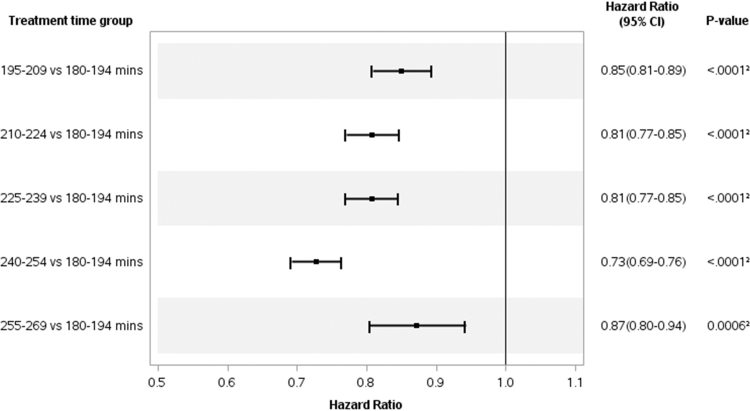
Adjusted mortality hazard ratios by treatment group. ∗Inverse probability of treatment weighting variables included age, sex, race, ethnicity, clinic geography, median household income, dialysis vintage, access type, primary insurance, residual kidney function, Kt/V, urea reduction ratio, interdialytic weight gain, ultrafiltration volume, pre- and post-dialysis blood pressure and weight, albumin, bicarbonate, calcium, hemoglobin, intact parathyroid hormone, phosphorus, sodium, white blood cell count, body mass index category, history of cancer, congestive heart failure, chronic obstructive pulmonary disease, cardiovascular disease, dementia, diabetes, hypertension, and peripheral arterial disease. CI, confidence interval.

Baseline confounders with missing data were handled using multiple imputations. All subsequent analyses were performed on the 20 imputed data sets. Using multinomial logistic regression, we estimated generalized propensity scores for each treatment time group. The model included baseline demographic, clinical, laboratory, and comorbidity covariates (Figure 2 footnote). The resulting predicted probabilities were merged with the marginal treatment distribution to compute stabilized inverse probability weights based on the probability of achieving the mean delivered treatment time of each group. The weights were truncated to the interval of 0.01 and 10 to limit the influence of extreme values. Absolute standardized mean differences were used to compare baseline covariates before and after inverse probability of treatment weighting (Supplementary Figure S2). For each covariate, the maximum imbalance across treatment groups versus the 180–194 minutes treatment group was calculated within each imputed dataset and then averaged across imputations.

The primary outcome of all-cause mortality was analyzed with a Fine-Gray proportional subdistribution hazards model,^6^ with estimates and standard errors pooled across imputations to obtain overall estimates, 95% confidence intervals, and *P*-values. Receipt of a kidney transplant, change in dialysis modality, and recovery of kidney function were treated as competing events in the model. Secondary outcomes included all-cause hospital admission rates and total hospital days. Hospitalizations < 24 hours were considered 1 hospital day. Any hospital admissions > 30 days were truncated to 30 days. Poisson regression was used to estimate pooled rate ratios for both hospital admissions and hospital days. Subgroup analyses were conducted by age (18–64, 65–80, and > 80), UF volume (< 2 L, 2–3 L, and > 3 L), sex (male and female), and baseline spKt/V (≤ 1.4 and > 1.4). We chose spKt/V > 1.4 because FKC uses this threshold to guide recommended adjustments in the dialysis prescription.

All data were extracted from the FKC clinical data warehouse and deidentified. Analyses were conducted using SAS Enterprise Guide version 7.1 (SAS Institute Inc, Cary, NC). The study was determined to be exempt under 45 CFR § 46.104(d)(4) by an independent institutional review board (New England Institutional Review Board [NEIRB], Needham, MA; WCG IRB Work Order #1-1801832-1).

## Results

**Figure 3:**
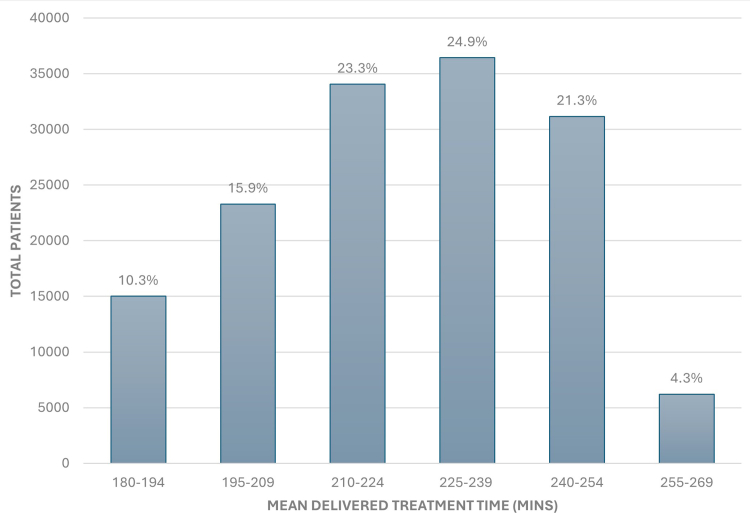
Distribution of patients by treatment time group.

After exclusions (Supplementary Figure S1), a total of 146,127 patients were included in the analysis. Overall, the mean delivered treatment time was 222 minutes. Patients were stratified into 6 treatment time groups based on their mean delivered treatment time, namely 180–194, 195–209, 210–224, 225–239, 240–254, and 255–269 minutes. The most frequently delivered treatment time was 225–239 minutes (25.0%), followed by 210–224 minutes (23.3%), 240–254 minutes (21.3%), 195–209 minutes (15.9%), 180–194 minutes (10.3%), and 255–269 minutes (4.3%) as shown in Figure 3. Selected baseline patient characteristics by treatment group are presented in Table 1. The complete list of baseline variables included in model adjustment is presented in Supplementary Table S1.

**Table 1:** Baseline characteristics of patients stratified by delivered treatment time

Characteristics	Total delivered treatment time (mins)					
	180–194	195–209	210–224	225–239	240–254	255–269
	(*n* = 15,000)	(*n* = 23,286)	(*n* = 34,050)	(*n* = 36,422)	(*n* = 31,159)	(*n* = 6210)
Age	66.6 ± 14.1	64.8 ± 14.1	64.7 ± 13.8	62.2 ± 13.4	62.6 ± 13.0	57.8 ± 12.1
Female sex	9665 (64.6)	12,511 (53.7)	15,490 (45.5)	12,932 (35.5)	8909 (28.6)	1013 (16.3)
Black race	4957 (33.1)	8314 (35.7)	11,689 (34.3)	13,928 (38.3)	11,141 (35.8)	2912 (46.9)
Hispanic ethnicity (*n* = 143,331)	2771 (18.5)	4429 (19.0)	6892 (20.2)	7103 (19.5)	6185 (19.9)	780 (12.6)
Vintage	5.0 ± 4.6	4.8 ± 4.4	4.5 ± 4.2	4.5 ± 4.1	4.2 ± 4.0	4.9 ± 4.2
BMI (*n* = 145,390)	26.3 ± 6.0	27.8 ± 6.5	28.6 ± 6.6	30.5 ± 7.2	31.5 ± 7.5	36.5 ± 8.5
Delivered treatment time	185.4 ± 4.9	202.6 ± 4.9	214.8 ± 4.6	232.4 ± 4.8	242.9 ± 3.4	260.4 ± 5.0
Pretreatment systolic blood pressure	151.3 ± 20.0	151.3 ± 19.9	150.5 ± 19.7	150.5 ± 19.8	149.6 ± 19.7	151.0 ± 20.4
Estimated dry weight	68.7 ± 17.0	74.2 ± 17.8	77.8 ± 18.6	85.7 ± 20.4	90.1 ± 22.1	110.1 ± 25.5
Interdialytic weight gain (*n* = 145,527)	2.5 ± 1.2	2.6 ± 1.2	2.7 ± 1.1	2.8 ± 1.2	2.9 ± 1.2	2.9 ± 1.2
Central venous catheter (*n* = 146,114)	3321 (22.1)	5244 (22.5)	7761 (22.8)	7907 (21.7)	6882 (22.1)	1281 (20.6)
Ultrafiltration rate (*n* = 145,706)	7.7 ± 3.3	7.5 ± 3.0	7.4 ± 2.8	7.2 ± 2.7	7.0 ± 2.6	6.7 ± 2.4
Single-pool Kt/V (*n* = 146, 126)	1.7 ± 0.3	1.7 ± 0.3	1.7 ± 0.3	1.7 ± 0.3	1.7 ± 0.3	1.6 ± 0.3
Serum albumin (g/dl) (*n* = 146,126)	3.8 ± 0.4	3.8 ± 0.4	3.9 ± 0.4	3.9 ± 0.4	3.9 ± 0.4	3.9 ± 0.4
Comorbidities						
Diabetes mellitus	8650 (57.7)	14,767 (63.4)	22,491 (66.1)	25,168 (69.1)	22,124 (71.0)	4564 (73.5)
CHF	3705 (24.7)	5833 (25.1)	8353 (24.5)	9674 (26.6)	8254 (26.5)	1818 (29.3)
Hypertension	13,225 (88.2)	20,824 (89.4)	30,210 (88.7)	32,704 (89.8)	27,732 (89.0)	5574 (89.8)
CVD	1392 (9.3)	2142 (9.2)	2964 (8.7)	3029 (8.3)	2369 (7.6)	428 (6.9)
PVD	1536 (10.2)	2424 (10.4)	3365 (9.9)	3733 (10.3)	3295 (10.6)	636 (10.2)
Cancer	835 (5.6)	1182 (5.1)	1771 (5.2)	1767 (4.9)	1521 (4.9)	247 (4.0)
Dementia	339 (2.3)	446 (1.9)	615 (1.8)	469 (1.3)	334 (1.1)	50 (0.8)
COPD	1675 (11.2)	2528 (10.9)	3432 (10.1)	3715 (10.2)	2952 (9.5)	641 (10.3)
Facility geography (*n* = 145,498)						
Rural	638 (4.3)	1000 (4.3)	1311 (3.9)	1675 (83.3)	1337 (4.3)	335 (5.4)
Suburban	1568 (10.5)	2428 (10.4)	3502 (10.3)	4248 (11.7)	3581 (11.5)	722 (11.6)
Urban	12,748 (85.0)	19,773 (84.9)	29,013 (85.2)	30,346 (4.6)	26,133 (83.9)	5140 (82.8)
Primary insurance (*n* = 146,126)						
Commercial	765 (5.1)	1421 (6.1)	2371 (7.0)	3255 (8.9)	2872 (9.2)	552 (8.9)
Medicare/Managed Medicare	12,435 (82.9)	18,794 (80.7)	27,048 (79.4)	28,104 (77.2)	23,995 (77.0)	4832 (77.8)
Medicaid/Managed Medicaid	1289 (8.6)	2010 (8.6)	2942 (8.6)	2738 (7.5)	2250 (7.2)	476 (7.7)
Other	511 (3.4)	1061 (4.6)	1689 (5.0)	2325 (6.4)	2041 (6.6)	350 (5.6)
Median income (*n* = 142,183)	84,318 ± 25,286	84,122 ± 24,355	84,662 ± 25,269	81,607 ± 23,228	80,934 ± 24,326	80,657 ± 22,831

BMI, body mass index; CHF, congestive heart failure; CVD, cardiovascular disease; COPD, chronic obstructive pulmonary disease; PVD, peripheral vascular disease.

The mean patient follow-up time was 315 days. Mean dialysis vintage was > 4 years, and few patients likely had residual kidney function. During the follow-up period, 20,729 patients died or withdrew from dialysis, 8840 left FKC, 572 recovered kidney function, 3547 changed dialysis modality, and 4142 underwent kidney transplantation. Compared with individuals in the 180–194 minutes group, individuals in groups with treatment times between 195 and 254 minutes had reduced mortality rates (Figure 2). In general, the association between longer treatment time and reduced mortality rate followed a dose-response pattern. Individuals in the 195–209, 210–224, 225–239, and 240–254 minutes groups had progressively reduced relative mortality rates of 15%, 19%, 19%, and 27%, respectively, when compared with individuals in the 180–194 minutes group. The longest treatment time group (255–269 minutes) was associated with reduced mortality rates compared with the 180–194 minutes group (13%).

A total of 170,475 hospital admissions occurred during the follow-up period. Patients in the 240–254 minutes group experienced a 19% (rate ratio: 0.81 [0.79–0.83]) reduced hospital admission rate, or 21.1 fewer hospital admissions per 100 patient-years, when compared with patients in the 180–194 minutes group. The next largest reductions were observed in the 225–239 and 210–224 minutes groups, with reductions of 13% (rate ratio: 0.87 [0.85–0.89]) and 12% (0.88 [0.86–0.89]), respectively. The 195–209 minutes group had a 7% reduced hospital rate relative to the 180–194 minutes group, (rate ratio: 0.93 [0.92–0.95]). The 255–269 minutes group had similar hospital admission rate as 180–194 minutes group (Table 2).

**Table 2:** Hospital admission rates (adjusted) by treatment group

Treatment group (min)	Hosps (pppy)	Rate ratio (CI)	Rate difference	Rate difference per 100 py	*P*-value
180–194	1.3	REF	REF	REF	REF
195–209	1.2	0.93 (0.92–0.95)	−0.068	−6.8	< 0.0001
210–224	1.2	0.88 (0.86–0.89)	−0.133	−13.3	< 0.0001
225–239	1.1	0.87 (0.85–0.89)	−0.140	−14.0	< 0.0001
240–254	1.1	0.81 (0.79–0.83)	−0.211	−21.1	< 0.0001
255–269	1.3	1.00 (0.97–1.03)	0.000	0.0	0.9946

CI, confidence interval; Hosps, hospitalization rate; pppy, per 100 person-yrs; py, person-yrs; REF, reference.

A similar pattern was observed across treatment groups when examining the number of days hospitalized (Table 3). Patients in the 240–254 minutes group had 20% fewer, or approximately 22 fewer days spent in the hospital per 100 patient-years compared with patients in the 180–194 minutes group (0.80 [0.80–0.81]). Patients in the 225–239 and 210–224 minutes groups had 15% fewer days (0.85 [0.85–0.86]), or approximately 16 fewer days per 100 patient-years. There was a 10% difference between the 195–209 minutes group compared with 180–194 minutes group. Finally, there was a small but statistically significant 5% increase in hospital days for the longest treatment time group.

**Table 3:** Hospital day rates (adjusted) by treatment group

Treatment group (min)	Hosp days (pppy)	Rate ratio (CI)	Rate difference	Rate difference per 100 py	*P*-value
180–194	8.4	REF	REF	REF	REF
195–209	7.6	0.90 (0.89–0.91)	−0.106	−10.6	< 0.0001
210–224	7.2	0.85 (0.85–0.86)	−0.158	−15.8	< 0.0001
225–239	7.2	0.85 (0.85–0.86)	−0.159	−15.9	< 0.0001
240–254	6.8	0.80 (0.80–0.81)	−0.221	−22.1	< 0.0001
255–269	8.9	1.05 (1.04–1.06)	0.047	4.7	< 0.0001

CI, confidence interval; Hosps, hospitalization rate; pppy, per 100 person-yrs; py, person-yrs; REF, reference.

**Figure 4:**
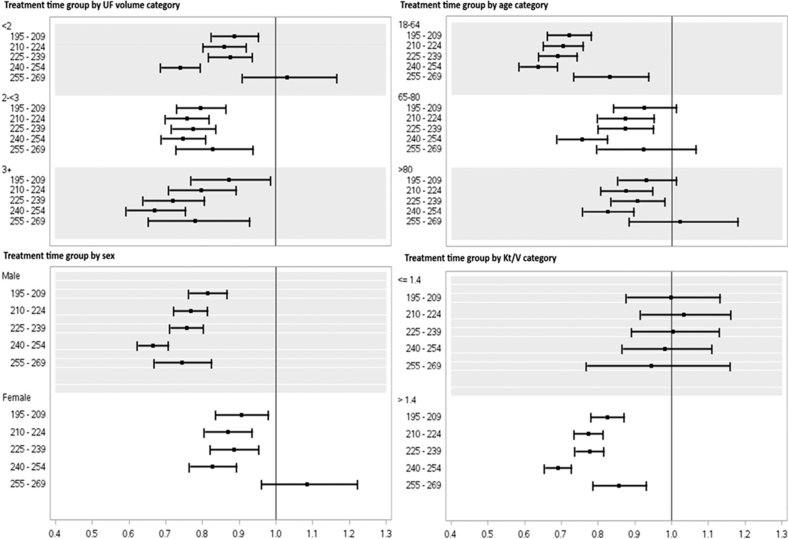
Subanalyses of adjusted mortality hazard ratios by treatment group. UF, ultrafiltration.

Subgroup analyses were conducted by age (18–64, 65–80, and > 80 years), UF volume (< 2 L, 2–3 L, and > 3 L), sex (male and female), and baseline spKt/V (≤ 1.4, > 1.4) (Figure 4). Patients aged 18 to 64 years in the 240–254 minutes group experienced the greatest reduction in mortality risk compared with patients in the same group aged 65 to 80 or >80 years (hazard ratio: 0.64 [0.59–0.69]). Males experienced a significant reduction in mortality risk in all treatment time groups, whereas females experienced significant reductions in in all groups but in the 255–269 minutes group. Patients with a mean baseline UF volume of ≥3 L experienced the greatest reduction of mortality risk but with increased variability compared with patients with an UF volume < 2 L and 2 to < 3 L. In addition, patients with a mean baseline spKt/V ≤ 1.4 did not experience any significant reduction in mortality risk.

## Discussion

Despite therapeutic innovations in extracorporeal kidney replacement therapies over the past few decades, the mortality rate for individuals receiving in-center hemodialysis for chronic kidney failure remains high.^7^ Extending hemodialysis treatment time to 240 to 254 minutes may represent a feasible, dependable, and reproducible means of improving patient survival. In this study of > 150,000 people receiving hemodialysis, we found that treatment times > 210 to 254 minutes were superior to shorter treatment times, and that the risk of mortality incrementally decreased with 15-minute increases in treatment time. In addition, we found reduced hospitalization rates and shorter hospital stays among individuals with treatment times approaching 240 minutes.

The benefit associated with longer treatment time was consistent across subgroups of sex and UF volume. Similarly, the findings were consistent across categories of age, except for the subgroup of patients aged > 80 years. This finding of an absence of benefit of extended treatment time on mortality for octogenarians confirms a similar finding from a large US dialysis organization cohort from 2007 to 2011.^8^ Individuals with spKt/V ≤ 1.4 did not show improvements in mortality with extended time on treatment, whereas individuals with spKt/V > 1.4 did.

We did not identify as large a reduction in the risk of mortality for patients with delivered treatment times between 255 and 269 minutes compared with the 240–254 minutes group (Figure 2). The reason for this lack of association is not clear; however, the cohort with this treatment time range tended to be younger (58 ± 12 years), with a mean estimated dry weight of 110 kg ± 22 kg, substantially higher than comparison patient groups, and a slightly higher percentage of these patients had an spKt/V of < 1.4 (19% vs. 11.2-12.6% in other comparison groups) (Table 1) This may imply residual bias in how treatment time was prescribed for those patients. In addition, this subset of patients only comprised 4% of the overall study population, and results may have been influenced by lower event numbers.

Previous studies have delivered a mixed verdict on the relationship between treatment time and mortality. The intent-to-treat analysis of the HEMO Study^9^ showed no evidence linking treatment time to mortality, though treatment time was tightly adjusted to achieve prespecified urea clearance thresholds,^10^ aiming for the shortest possible treatment time. Although a secondary analysis of the HEMO Study, treating time as a Cox-dependent covariate did show an association between shorter treatment time and higher mortality risk, these results may have been confounded by “dose targeting bias,” whereby the observed adverse mortality effects of shorter treatment times are magnified by setting higher treatment times as the baseline comparison group.^11^ The methods used for modeling treatment time may impact whether and to what extent treatment time is associated with mortality in observational data. For example, Brunelli *et al.*^12^ found no association between treatment time and mortality when treatment time was treated as a “baseline” measurement. However, when treatment time was specified as a time-varying exposure and time-dependent confounders were updated using a marginal structural analysis approach, treatment times ≤ 180 and 181–239 minutes, compared with the 240–254 minutes group, associated with higher risks of mortality.^12^

Longer treatment time has been associated with improved urea clearance as measured by threshold Kt/V, and lower interdialytic weight gain, each of which has been associated with improvement in survival. Controlling for achieved Kt/V, shorter treatment time (< 240 minutes) and, separately, larger interdialytic weight gain (> 3 kg) has been associated with higher mortality, suggesting a partially independent influence of treatment time on mortality.^13^ Concerns about potential confounding from body size in these studies were partially addressed by a separate analysis with matching on body size, which found an association between longer treatment time and a reduced mortality rate.^14^

At a conceptual level, extending treatment time is straightforward. However, operationalizing it at scale has proven challenging. Among the less well-recognized effects of the SARS-CoV-2 pandemic is the reduction in dialysis treatment time in the US. In 2023, only 28% of US hemodialysis patients had a treatment time ≥ 4 hours.^4^ Both patient and provider reluctance to extend time on treatment likely factor into this trend.^15^ Limited uptake of the intervention of hemodialysis initiation with a treatment time ≥ 255 minutes in the Time to Reduce Mortality in End Stage Kidney Disease trial support this hypothesis. Given the low probability of conducting another sufficiently powered randomized clinical trial examining the effects of extended treatment time on mortality and other adverse outcomes, guidance about optimal treatment times must be ascertained from the results of large cohort studies. Our study findings of associations between longer treatment times and better patient survival, reduced hospitalization rates, and shorter hospital length of stay suggest a need to develop educational and/or operational interventions to overcome patient-, provider-, and facility-level barriers to extending hemodialysis treatment times.

The strengths of this study include its large, contemporary cohort that is representative of the broader US hemodialysis patient population. Interpretation of study findings should consider limitations of the data. This is a retrospective, observational study, and we cannot rule out influence from selection bias or residual confounding (e.g., from unmeasured differences between the exposure groups such as residual kidney function and social determinants of health) despite our efforts to control for key confounders in the analysis. This is a perennial challenge with using observational data to parse out the contributions of discrete facets of the dialysis treatment prescription to outcomes.^16^ In addition, we did not have accurate measures of residual kidney function and related confounding may remain. However, the mean time on dialysis for patients in the cohort was > 4 years (Table 1), and previous studies suggest that the majority of individuals receiving hemodialysis have lost consequential residual kidney function by this timepoint.^17^

In conclusion, our findings provide additional observational data supporting an association between longer treatment time and better outcomes. These findings suggest that it may be advantageous to extend treatment time for most patients, preferably to a minimum of 240 minutes, but even to just > 210 minutes for individuals with prescribed treatment times < 210 minutes. However, like with all interventions, shared decision-making that considers individualized risk-benefit assessments and patient preferences should be used to guide changes in prescribed hemodialysis treatment time.

## Disclosure

In the last 2 years, JEF received consulting fees from Aquapass and Fresenius Medical Care, North America and royalties from UpToDate and served on a scientific advisory board for the NIDDK and a Data and Safety Monitoring Committee for Veterans Affairs. LHF and RL are employees of Employee of Renal Research Institute LLC, a wholly owned subsidiary of Fresenius Medical Care Holdings Inc. LHF owns stock in Fresenius Medical Care. BEH is a full-time employee of Fresenius Medical Care. BEH has equity stake and/or consulting arrangements with Interwell Health, Revalia Bio, Nephronomics, and Klinrisk.

## Supplementary Material

Supplementary File (PDF) **Figure S1.** Patient Selection. **Figure S2.** Absolute standardized mean differences before and after IPTW. **Table S1.** Baseline characteristics by treatment time group. STROBE Checklist.

## Footnotes

Supplementary File (PDF)
**Figure S1.** Patient Selection. **Figure S2.** Absolute standardized mean differences before and after IPTW. **Table S1.** Baseline characteristics by treatment time group. STROBE Checklist.
